# Ethically utilising COVID-19 host-genomic data

**DOI:** 10.1038/s41525-021-00194-9

**Published:** 2021-05-10

**Authors:** Christopher Gyngell, John Christodoulou, Julian Savulescu

**Affiliations:** 1 Murdoch Children’s Research Institute, Melbourne, Australia; 2 Department of Paediatrics, University of Melbourne, Melbourne, Australia; 3 Faculty of Philosophy, Oxford Uehiro Centre for Practical Ethics, Oxford, UK

**Keywords:** Genetics research, Genetics

## Abstract

Genetic variants that influence susceptibility to COVID-19 have recently been identified. In this manuscript, we identify and discuss some of the ethical and practical issues raised by these studies. We first outline the ethical case for providing COVID-19 susceptibility testing to healthcare workers, as well as highlighting risks associated with privacy and discrimination. We then argue that the existence of genetically susceptible individuals has implications for the ethical conduct of COVID-19 human challenge trials. Finally, we discuss the ethical issues that could arise from other COVID-19 host–genome interactions, including the prospect of personalized vaccines.

## Introduction

A number of genetic variants that influence susceptibility to COVID-19 have been identified. This includes variants associated with known disorders of immunity, variants associated with common polymorphisms, and variants uniquely associated with risk of COVID-19^1^. Several large initiatives such as *the COVID Human Genetic Effort* (https://www.covidhge.com), and *The COVID-19 Host Genetics Initiative* (https://www.covid19hg.org) collectively aim to analyse how our genome influences our response to SARS-CoV-2 infection. One of the reasons these studies are useful is that they help uncover the biological mechanisms which are important to COVID-19 outcomes and generate hypotheses for treatment. Another stated motivation of these studies is to one day be able to “identify individuals who have either an unusually high or low risk of developing severe COVID-19”^2^. Some researchers have described this research as a step “towards risk stratification, personalized treatment plans, therapeutic, and vaccine development and deployment”^2^. The ethical implications of using COVID-19 data in this way, have to date, not been scrutinized.

Information about genetic predisposition to severe COVID-19 could be of high personal utility for individuals, as well as help policymakers target resources to those who are the most vulnerable. At the same time, genomic testing raises ethical concerns, relating to privacy, and discrimination.^3,4^ In this manuscript, we outline some host–genome interactions that have been, or may be, uncovered for COVID-19 and discuss some of the opportunities and concerns they raise.

### Severity variants

One feature of COVID-19 is its high interpersonal variability, with some infected individuals being completely asymptomatic and others developing severe respiratory failure. Even after taking demographic factors such as age and other risk factors such as pre-existing cardiovascular disease, obesity, diabetes mellitus, chronic liver or renal disease, malignancy into account, there remains significant variation in COVID-19 severity. Individual genetic differences contribute to this variation. Whole-genome sequencing of individuals who have been infected with the SARS-CoV-2 virus found those, who develop life-threatening symptoms were eight times more likely to have a loss of function variant in genes which influence the regulation of type-1 interferons, an important component in the innate immune response^5^. Another study identified a region on chromosome 3 that is associated with a 1.7 times greater risk of requiring mechanical ventilation after infection with SARS-CoV-2, as well as earlier age at hospitalization^6^. This region has then been mapped to variants first introduced into the human gene pool via Neanderthal interbreeding 60,000 years ago, and is relatively common, carried by ~30% of people living in South Asia and ~8% of people living in Europe^7^. A study published in *Nature* replicated these earlier associations, and identified additional novel variants that increase risk of severe COVID-19, including in a gene cluster that encodes antiviral restriction enzyme activators^8^.

Genomic information could be combined with other medical and demographic data to produce overall measures of COVID-19 risk. Recently, personal genomics company 23andMe, developed a COVID-19 severity predictor tool (https://you.23andme.com/covid19/). While this tool currently only uses demographic data and medical history, 23andMe has conducted genome-wide associations studies, which have identified various risk variants for severe COVID-19. It would only be a short step for 23andMe to incorporate genomic data into their COVID-19 severity calculator.

In the next section, we will consider some possible applications of using genomic tests, either alone in combination with lifestyle data, to help inform overall risk considerations. Our goal is to identify both some of the opportunities of host-genomic testing, and the ethical concerns raised.

### Healthcare workers

Protecting essential workers—who provide basic services such as healthcare, food, and logistics—is critical during pandemics. Essential workers, particularly healthcare workers, have been especially impacted from the COVID-19 pandemic. As of September 2020 more than 7000 healthcare workers (HCWs) have died due to COVID-19^9^. The median age of death for HCWs from COVID-19 is 56.2^10^, more than 20 years younger than in the general population. It is highly likely that genetic susceptibility to COVID-19 was a factor in some of these deaths, given the frequency of variants already identified.

If we could use genomic testing as a way of predicting one’s risk of COVID-19, we would have ethical reasons to make it available to HCWs out of respect for their autonomy. HCWs are often considered to have an obligation to treat patients, even if doing so exposes them to risks such as being infected by COVID-19. For example, the American Medical Association (AMA) has stated that physicians have an obligation to “provide urgent medical care during disasters”, an obligation that holds “even in the face of greater than usual risk to physicians’ own safety, health or life.”^11^. While HCWs in some jurisdictions may not have an explicit professional obligation to provide care to others in the face of personal danger, many choose to do so, as do many other essential workers. This enhances, rather than diminishes, our reasons to provide them with information about personal risk. When individuals are making high-stakes decisions and are exposed to harm, they have greater claims on information that can help inform these decisions.

There are also beneficence-based reasons to offer HCWs susceptibility testing. Given the pivotal role played by health systems, especially in pandemics, disruptions due to HCW illness and death can cause widespread harm. Suppose genetic testing enabled healthcare systems to be structured better so that the most vulnerable workers are protected from exposure. In that case, this could allow healthcare systems to operate more efficiently and may result in wide benefits. However, this would require us to allocate healthcare workers into different care settings according to their genetic background which raises ethical concerns.

One of the worries of genetic testing is discrimination. Imagine genetic testing is offered to HCWs as a way of informing workforce allocation, and some are found to be of high risk for severe COVID-19. We can foresee situations where those workers fail to get their employment contracts renewed, or are otherwise disadvantaged, as a result of this test. While such clear cases of discrimination based on genotype are illegal in many parts of the world (including in the US through the *Genetic Information Non-discrimination Act)*, genetic testing can also lead to more subtle forms of discrimination and coercion. Those identified as being resistant to severe COVID-19 may feel more pressure to undertake high-risk activities and shield other workers from infection. Failure to act in these altruistic ways may lead to judgment from others. Far from empowering HCWs to make informed decisions, genetic testing could end up creating additional social expectations which constrain their freedom of choice.

In sum, if genomic tests can help predict COVID-19 severity there is an ethical case that we should make them available to HCWs, and others in high-risk occupations, out of respect for their autonomy. However, before the implementation of any testing, it will be important to carefully analyse the potential implications for workplace discrimination and privacy. Further ethical and legal analysis of offering genetic testing to essential workers, as well as more empirical research on attitudes of HCWs to genetic testing is needed as we uncover more about the genetic factors that contribute to COVID-19 susceptibility.

### Human challenge trials

Over 30,000 people have already volunteered to participate in COVID-19 human challenge trials and numerous trials are planned^12^. Challenge trials deliberately expose participants to infection, and in doing so can rapidly increase the speed of vaccine development. The COVID-19 pandemic has had a devastating effect on the world through both its health and economic impacts. Even a small increase in the rate at which vaccines are developed and employed could save thousands of lives and reduce the economic impact of COVID-19.

Human challenge trials are ethically controversial, mainly because they expose healthy volunteers to potentially severe disease. Nonetheless, many ethicists have endorsed COVID-19 human challenge trials, provided they meet stringent conditions^13^. The World Health Organization (WHO) supports challenge trials provided they are underpinned by “rigorous informed consent” and use participant selection criteria that limit and minimize risk^14^. For these reasons, it has been recommended that challenge trials for COVID-19 initially be limited to young adult volunteers.

However, the same ethical principles that limit challenge trial participants to young adults also imply we should limit participation to those who do not carry genetic susceptibilities to severe COVID-19. The World Health Organization recommendations state that “participants who face a higher expected magnitude of harm should be excluded”, and this would apply to those who have a genetic predisposition to severe disease. Therefore, it may be a breach of current guidelines to run challenge trials without testing participants for underlying genetic susceptibilities.

If we exclude those who have a genetic susceptibility to COVID-19 from participating in challenge trials, it may reduce the generalizability of the results. However, it is hard to see how excluding those with genetic susceptibility to COVID-19 will reduce the generalizability of the results more than excluding those aged over 30, which is the current practice. Additionally, functional genetic studies could help inform estimates of generalizability. For example, some variants which predispose to severe COVID-19 have been shown to play a role in innate immunity, which may indicate they are not likely to affect an individual’s response to vaccine.

Furthermore, there may be ways we can use genomic testing to improve the generalizability of results. All challenge trials must balance the expected risk to participants against the generalizability of the results^14^. For COVID-19, young adults have been proposed as participants in challenge trials, as they have a low risk of becoming very sick due to exposure to SARS-CoV-2. However, it is older adults who are generally prioritized for COVID-19 vaccinations. Ideally, vaccines would be tested on older adults who are at low risk of severe disease due to SARS-CoV-2. Through genetic testing, it might be possible to identify such individuals.

### Resistance variants

It has long been established that some individuals carry genomic variants that make them immune to particular infectious diseases. One of the most well-known examples is the *CCR5*-Delta32 polymorphism. CCR5 codes for a cell receptor found in immune cells. Some people have a 32 base-pair deletion in this gene (called the delta32 variant), which alters the receptor in such a way that the HIV virus cannot enter the cells, and this protects against AIDS^15^. Variants conferring cellular resistance to norovirus^16^, and malaria^17^ have also been identified.

The next step of the *COVID Human Genetic Effort* is to undertake genome sequencing of individuals who have never contracted COVID-19, despite repeated exposures. These studies will look for variants that make people resistant to infection from SARS-CoV-2, for example, by making it impossible for the virus to enter one’s cells.

What would the ethical implications of identifying such variants be? If some people are entirely resistant to SARS-CoV-2, they pose no risk of spreading the virus to others. This has implications for the ethical justification of public health policies that have been adopted as a response to COVID-19. Many governments have imposed forced lockdowns on entire communities that restrict everyone’s ability to leave their home for non-essential purposes, as well as imposing mandatory quarantine periods for travelers. These policies exact a cost on individuals by restricting their freedom of movement. Significantly, these costs disproportionately harm members of a community who are already the worst-off, such as those with insecure employment, and those without financial and personal safety nets.

Lockdowns and quarantines are often justified on the basis that they are the least restrictive way possible to prevent outbreaks and thus promote a public good^18^. Because we cannot know who in a community is carrying the virus, allowing the free movement of any individual increases the net risk to all. However, if it becomes possible to identify individuals who genuinely pose no threat to others, this justification will not apply to them. Restricting the movement of people who pose no risk to others does not promote a public good, and thus imposes a cost on individuals for no benefit. If some of these resistant individuals are already badly off, this would be a significant injustice.

The possibility of identifying resistance variants for COVID-19 raises similar questions to those raised by the prospect of “immunity passports”, a hypothetical proposal to allow more freedom of movement for those who have been infected with COVID-19 and may now be immune. Just like “immunity passports”, “resistance passports” may exacerbate existing inequalities if only the relatively wealthy can access them. If variants are identified which make people resistant to COVID-19, it will be essential to consider how testing for these variants could be deployed in ways that reduce, rather than exacerbate, existing social inequalities.

Furthermore, if some individuals are granted special exemptions from lockdowns and quarantines, it might encourage other individuals to engage in more risky behavior, and consequently lead to a greater spreading of the virus. Part of the effectiveness of public health measures, such as social distancing, wearing masks, and lockdowns, depend on a sense of solidarity. Individual personal sacrifices are worth it because others are also making sacrifices for the greater good. If people who are at low personal risk of disease stop making sacrifices, this may undermine the collective camaraderie essential for successful public health measures.

These ethical considerations are important to carefully think through, not only for the purposes of responding to the COVID-19 pandemic, but also future pandemics where some individuals are genetically resistant.

### Personalized vaccines

Further research into COVID-host genomics may yield other types of information that can be used to reduce the impact of the COVID-19 pandemic. As of March 2021, there are several approved vaccines for COVID-19 with varying mechanisms and effectiveness. Dozens more candidates are in clinical trials. With over 7 billion doses of vaccine needed, it is now widely recognized that many different vaccines are required to achieve worldwide resistance to COVID-19^19^. Numerous studies have shown that one’s genome influences responses to vaccination and genetic heterogeneity has been identified as a primary obstacle in offering safe vaccines to the public^20^. It could be that some vaccines for COVID-19 will have variable effects that are at least partly mediated by one’s genome. If these genetic differences are documented and understood, it could increase our understanding of the adverse events associated with vaccination and the efficiency at which vaccine candidates are safely administered to the public. Future pandemics are likely to take place in the context of more developed and widespread genomic technologies^21^, and considerations regarding how genomic information can be used to inform vaccine delivery will be more crucial.

Vaccine development represents a distinct ethical challenge. Vaccines are a medical intervention designed to be used by millions of healthy people. A founding principle of medical ethics is “non-maleficence”, which stresses the special obligation to ensure people having medical intervention are not made worse than they otherwise would have been. As people who are receiving vaccines are generally healthy, the intervention raises special concerns regarding non- maleficence. This makes rigorous safety trials essential.

However, in a pandemic, delays in vaccine development cost lives. As of February 2021, more than 10,000 people a day are dying from COVID-19. Every single day an effective vaccine is delayed costs lives. Reasons of beneficence, to do the most good, compel us to ensure a quick rollout.

We can imagine cases where genetic associations could help us resolves this dilemma. Imagine two vaccine candidates are developed A and B, but each cause adverse events in roughly 1% of cases. This would be far too high a rate of adverse events for the vaccines to be administered to the general public. But imagine that these adverse events are non-overlapping, so that if one has a severe reaction to A, then they will not have one to B and vice versa. Further, imagine that these effects could be predicted by a genetic test. While this is a hypothetical case, it is not unrealistic. Genetic variants in the *IRF1* gene can predict adverse events due to the smallpox vaccine, and adverse events due to the yellow-fever and influenza vaccines have also be associated with genetic polymorphisms^22^. It is a crucial time to consider how we might go about using host-genome data to better use vaccine resources.

An interesting question for cases like those described above is whether we have reasons to go beyond merely offering genetic tests to enable people to receive vaccinations, but to make genetic tests mandatory. Recent research has identified obstacles to obtaining effective vaccination levels for any COVID-19 vaccine, and recent US polls “suggest only 3 in 4 people would get vaccinated if a COVID-19 vaccine were available, and only 30% would want to receive the vaccine soon after it becomes available.”^23^. To counter vaccine hesitancy, it has been suggested that we should make vaccines mandatory—or offer financial inducements—to ensure we achieve effective vaccination rates. Compulsory vaccination has been seen as ethically justified where the following conditions are met^24^:

1. There is a grave threat to public health.
2. The vaccine is safe and effective.
3. Mandatory vaccination has a superior cost/benefit profile compared to other alternatives.
4. The penalties associated with non-compliance are proportionate.

When conditions 1 and 3 hold, and genetic tests help achieve condition 2, then there are also good reasons to make genetic testing mandatory.

There are objections to mandatory measures in public health such as vaccination. Some claim mandatory vaccination is not permissible unless there is a serious risk of harm to others^25^. The Nuffield Council of Bioethics (amongst others) have argued we should always adopt the Least Restrictive Alternative^26^. Another popular argument against mandatory vaccination is that it can backfire by eroding people’s trust. There will likely be strong opposition to making any type of genetic testing mandatory, even if it helped improve vaccine safety and efficiency. Making such testing mandatory could erode public trust in vaccinations more generally.

As genomics becomes more and more integrated into medicine, the idea of using genetic tests to guide vaccination could become accepted. This shows the importance of continued public engagement on the use of genomics in medicine.

**Table 1:** Summary of the potential opportunities and concerns of potential COVID-19 host-genome testing applications.

Application	Opportunities	Ethical concerns
Healthcare workers	Promote autonomy, protect vulnerable workers	Discrimination, privacy, and stigmatization
Challenge trials	Protect vulnerable participants	Reduce generalizability of results
Amend quarantine lockdown conditions	Avoid unnecessary restrictions on movement	Reduce communal camaraderie and solidarity.
Personalized vaccines	More effective distribution of vaccines	Reduced trust

## Conclusion

The COVID-19 pandemic is unfolding in a world where the tools of genomics are increasingly used in medicine. There are opportunities to use host-genomic information to lessen the impact of the pandemic by shielding HCWs who are most vulnerable, as well as better informing the design of human challenge trials. Host-genomic information may help us better predict the effects of vaccination and increase the efficacy at which vaccines are developed and employed. In order to reap these benefits, we must confront the ethical, legal, and social challenges posed by host-genomic data (see Table 1). Genetic testing may lead to discrimination and subtle forms of coercion and must be accompanied by strong privacy protections. Furthermore, the identification of people who are resistant or at low risk of severe COVID-19, risks undermining communal camaraderie that has been essential to the success of public health initiatives like lockdown. As more COVID-19 host-genomic data is made available, more thought must go into how to utilize it ethically.
